# Improved Dried Blood Spot-Based Metabolomics: A Targeted, Broad-Spectrum, Single-Injection Method

**DOI:** 10.3390/metabo10030082

**Published:** 2020-02-27

**Authors:** Kefeng Li, Jane C. Naviaux, Jonathan M. Monk, Lin Wang, Robert K. Naviaux

**Affiliations:** 1 The Mitochondrial and Metabolic Disease Center, School of Medicine, University of California, San Diego, CA 92103, USA; jnaviaux@health.ucsd.edu (J.C.N.); liw004@health.ucsd.edu (L.W.); 2 Department of Medicine, School of Medicine, University of California, San Diego, CA 92103, USA; jonathan.m.monk@gmail.com; 3 Department of Neuroscience, School of Medicine, University of California, San Diego, CA 92103, USA; 4 Department of Pediatrics, School of Medicine, University of California, San Diego, CA 92103, USA

**Keywords:** metabolomics, dried blood spots, targeted, broad-Spectrum, MELAS

## Abstract

Dried blood spots (DBS) have proven to be a powerful sampling and storage method for newborn screening and many other applications. However, DBS methods have not yet been optimized for broad-spectrum targeted metabolomic analysis. In this study, we developed a robust, DBS-based, broad-spectrum, targeted metabolomic method that was able to measure over 400 metabolites from a 6.3 mm punch from standard Whatman 903^TM^ filter paper cards. The effects of blood spot volumes, hematocrit, vacutainer chemistry, extraction methods, carryover, and comparability with plasma and fingerstick capillary blood samples were analyzed. The stability of over 400 metabolites stored under varying conditions over one year was also tested. No significant impacts of blood volume and hematocrit variations were observed when the spotted blood volume was over 60 µL and the hematocrit was between 31% and 50%. The median area under the curve (AUC) of metabolites in the DBS metabolome declined by 40% in the first 3 months and then did not decline further for at least 1 year. All originally detectable metabolites remained within detectable limits. The optimal storage conditions for metabolomic analysis were −80 °C with desiccants and without an O_2_ scavenger. The method was clinically validated for its potential utility in the diagnosis of the mitochondrial disease mitochondrial encephalomyopathy, lactic acidosis, and stroke-like episodes (MELAS). Our method provides a convenient alternative to freezing, storing, and shipping liquid blood samples for comparative metabolomic studies.

## 1. Introduction

Metabolomics is emerging as an important tool in clinical chemistry to identify biomarkers, find new roles of metabolism in disease, and discover novel metabolic drug targets [1,2,3]. Blood-based samples, including serum and plasma, are probably the best studied biofluids for metabolomics [4]. However, serum and plasma preparation requires laboratory healthcare professionals and stringent storage conditions to avoid metabolite degradation. Dried blood spots have been used for newborn screening in millions of children since the 1960s [5]. In this paper, we explore the utility of dried blood spot (DBS) analysis in metabolomic studies.

Samples prepared and stored as dried blood spots on standardized filter paper cards may be an attractive alternative to storage, shipment, and analysis of conventional liquid venous blood samples for many studies. One advantage of dried blood spots is that quantitative results for many targeted molecules can still be obtained within relatively large margins of variation in the original amount of blood and hematocrit spotted. This occurs because of reproducible spreading of the sample by the fibers of standardized filter paper. For example, a 1/8th inch (3.175 mm) diameter punch from a standard dried blood spot on Whatman 903^TM^ paper contains the residue from 3.1 µL of blood [6], without the need for great volumetric precision in the amount of blood applied as long as the amount is sufficient (50–100 µL; 1–2 drops) to fill at least 90% of the 12-mm dotted circles inscribed on the cards [7].

Fingerstick lancet and pipetting from vacutainer tubes of collected venous blood are two common methods for DBS preparation. With regard to DBS prepared by fingerstick, it is minimally invasive, requires a small blood volume, and the samples can be collected by the patients themselves and sent to the assigned labs by regular mail with low cost [8]. With regard to DBS prepared by health professionals by pipetting from blood collected in vacutainer tubes, samples stored as DBS may be useful for many applications. The benefits of dried blood spots are of particular interest for comparative metabolomics studies conducted in remote locations, and those having large cohorts from multiple collaborating centers [9]. In recent years, due to the above benefits, DBS have also been increasingly used in various other fields, such as therapeutic drug and abused substances monitoring [8,10], pharmacokinetics [11], proteomics [12], lipidomics [13,14], and metabolomics [15,16,17,18].

Although metabolomic analysis of DBS samples has been reported in a few studies, there is still a lack of systematic optimization of the specific protocols for DBS preparation and storage for metabolomics. In addition, previous DBS-based metabolomics methods often require multiple injections and platforms to increase the metabolome coverage. These methods have only measured a small number of metabolites [13,16]. A single liquid chromatography-tandem mass spectrometry (LC-MS/MS) platform targeting a large number of metabolites with a broad range of chemical classes is highly desirable to fulfill different application requirements, reduce the cost, and increase the productivity of DBS-based metabolomics. Moreover, the stability of the whole blood metabolome in DBS under different storage conditions remains largely unknown except for some routinely analyzed metabolites in newborn screening, such as acylcarnitines and amino acids [4].

MELAS (mitochondrial encephalomyopathy, lactic acidosis and stroke-like episodes) is a mitochondrial disorder with symptoms that can also include headache, fatigue, diabetes, and hearing loss [19]. Nearly 80% of MELAS patients have an adenine-to-guanine transition mutation at position 3243 of their mitochondrial DNA (A3243G) located in the mitochondrial tRNA-leucine (UUR) gene [20]. The metabolomic analysis of disease-specific metabolic fingerprints in the DBS of MELAS patients might provide useful information for the diagnosis and potential treatment targets of MELAS.

In this study, we developed a DBS-based broad-spectrum, targeted metabolomic method and performed the systematic optimization of critical steps in the workflow. The effect of variations in blood spot volumes, extraction methods, carryover, the comparability with other types of blood samples, and the potential influence of hematocrit variations were analyzed. Additionally, the stability of over 400 metabolites stored as DBS samples under three different storage conditions was analyzed periodically for up to one year. The analytical reproducibility and potential clinical utility were demonstrated with DBS samples prepared from 12 pediatric patients with MELAS and 12 age- and sex-matched controls.

## 2. Materials and Methods

### 2.1. Informed Consent

**Figure 1:**
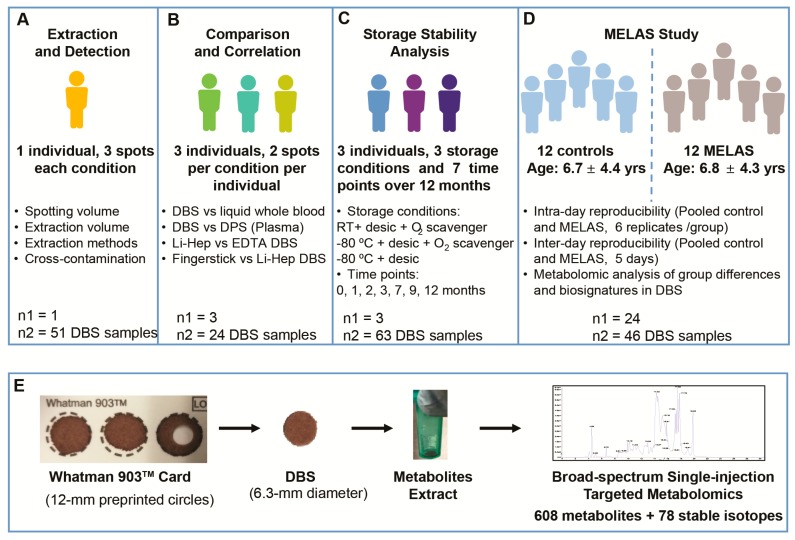
Overview of the study design and clinical cohorts. (**A**) Extraction and carryover. (**B**) Sample types. (**C**) Storage stability. (**D**) MELAS study. (**E**) DBS extraction and analysis.

The study was conducted with signed informed consent under the approved (2/24/2014) University of California, San Diego (UCSD) institutional review board (IRB) protocol #140072. The elements and design of this study are illustrated in Figure 1A–D.

### 2.2. DBS and DPS (Dried Plasma Spot) Preparation

Whole venous blood was collected using Li-heparin vacutainer tubes (Becton-Dickinson #367884, Franklin Lakes, NJ, USA) or ethylenediaminetetraacetic acid (EDTA) vacutainer tubes (Beckton-Dickinson #367861) mixed by inversion 10 times. Plasma was obtained by centrifugation of the whole blood at 900× g for 10 min at room temperature (RT). The DBS samples were prepared by pipetting 70 µL of Li-heparin whole blood (Li-hep DBS) or EDTA whole blood (EDTA DBS) on to Whatman 903^TM^ paper cards (GE Healthcare, #10534612, Chicago, IL, USA). Dried plasma spot (DPS) samples were prepared by spotting 40 µL of Li-hep plasma onto Whatman 903^TM^ paper cards. Additionally, blood via fingerstick was taken using a sterile lancet and directly spotted onto the circles of a paper card (Fingerstick DBS). One to two drops was sufficient to fill the 12-mm inner diameter of the dotted circle when applied in the center. The DBS cards were air-dried at room temperature (RT) for 24 h. The disks were punched out from the DBS using a ¼ inch diameter (6.3 mm) one-hole puncher (ACCO, Catalog: 74005). Punching from an empty area of the sampling card (Blank disks) was performed in between every sample.

### 2.3. Method Optimization

Blood spotting volumes on Whatman 903^TM^ paper cards were optimized. Five blood spotting volumes (40, 50, 60, 70, and 80 µL) were tested. The volumes of extraction buffer (100, 200, 300, 400, 500 µL) and the extraction methods (20 min on ice, overnight at RT, and sonication for 5 min on ice) were also optimized. The potential cross-contamination was assessed by comparing the level of a metabolite in a DBS sample with the levels in the following 2 blank paper punches. Each condition was measured in triplicate.

### 2.4. Sample Type Comparisons

Samples were collected from 3 subjects with duplicates for each condition (Figure 1B). Sample comparisons included Li-hep DBS versus liquid whole blood from lithium-heparin venous blood, DBS versus DPS, DBS obtained from Li-heparin versus EDTA vacutainer tubes, and fingerstick DBS versus Li-heparin DBS.

### 2.5. Stability Analysis

DBS samples from 3 individuals were collected at the baseline (after drying for 24 h), 1, 2, 3, 7, 9, and 12 months (Figure 1C). The following storage conditions were tested: (1) Room temperature (RT) with desiccant and an oxygen scavenger (RT + desic + O_2_ scavenger), (2) −80 °C with desiccant (−80 °C + desic); and (3) −80 °C with desiccant and an oxygen scavenger (−80 °C + desic + O_2_ scavenger). In each case, the DBS was placed in a light-tight, metalized Mylar bag (PackFreshUSA, Cat. #MGB50, Ontario, CA, USA) along with two desiccant packs (1.5 g/pack), with or without one bag of oxygen scavenger (300 cc, PackFreshUSA, Cat. #F3C1C, ferrous iron) and was then heat sealed.

### 2.6. Hematocrit Effects

Plasma and whole blood samples from the same individual were mixed to obtain hematocrit levels ranging from 0% to 50%. A total of 70 µL of the mixture was spotted on Whatman 903^TM^ paper cards and dried for 48 h before analysis. Three punches per hematocrit level were collected for further analysis.

### 2.7. Metabolite Extraction

The 6.3 mm disk punch was transferred to a 2 mL tube and 200 µL of extraction buffer consisting of prechilled MEOH-ACN-H_2_O (40:40:20, v/v/v), and custom-synthesized stable isotope internal standards were added. The list of internal standards has been previously described [21]. After incubation on ice for 20 min, it was centrifuged at 16,000 g for 10 min at 4 °C. The supernatant was transferred to a new tube and stored at −80 °C until analysis (Figure 1E).

### 2.8. Metabolomic Analysis

Samples were analyzed on a Shimadzu LC-20AD high-performance liquid chromatography (HPLC) system coupled with a SCIEX Qtrap 5500 mass spectrometer (LC-ESI-MS/MS) using a broad-spectrum targeted metabolomic method as previously described [21]. Briefly, metabolites were separated on a polymer-based NH_2_ column (250 × 2 mm, 4 µm, Asahipak NH_2_ P-40 2E, Showa Denko America, Inc., New York, NY, USA) in hydrophilic interaction liquid chromatography (HILIC) mode. A total of 622 endogenous metabolites and 78 stable isotope internal standards were targeted in a single injection [21]. The peaks were manually checked in Multiquant 3.0 (Sciex) and the peak area was exported for further analysis. The spiked stable isotope internal standards were used to monitor the metabolite extraction and MS detection.

### 2.9. Clinical Application in MELAS

Whole venous blood was collected from 12 MELAS patients, ranging in age from 0.3 to 11.6 years, and 12 age and sex-matched controls using Li-Heparin vacutainer tubes. The DBS samples were prepared by pipetting 70 µL of whole blood on to Whatman 903^TM^ paper cards (Figure 1D). The DBS samples were stored at RT with desiccant and were analyzed within a week after DBS preparation using the metabolomic method described above. The patients’ characteristics are listed in Table S1. The reproducibility of the assay was evaluated using 6 replicates of the pooled MELAS and control DBS extract analyzed on each of the 5 days [22]. The mean intra-assay, inter-assay, and total relative standard deviations (RSDs) for each pool were calculated [22].

### 2.10. Bioinformatic and Statistical Analysis

The data were expressed as mean ± SD unless otherwise indicated in the figure legends. A Student’s *t*-test or one-way ANOVA followed by Tukey’s test was used to compare the group differences. *p* < 0.05 was considered as statistically significant. The correlations were analyzed by Spearman’s rank correlation. Frequency distribution analysis was applied to show the range of RSDs for all detected metabolites.

Metabolomic analysis was performed in MetaboAnalyst 4.0 (https://www.metaboanalyst.ca/). Briefly, the mass chromatographic areas under the curve (AUCs) were log2-transformed, scaled by the SD of each metabolite, and analyzed by partial least squares discriminant analysis (PLS-DA). Metabolites with the highest separation power in PLS-DA were ranked by variable importance in projection (VIP). VIP scores > 1.5 were considered significant. Sets of 3–6 metabolites were selected manually from top 50 significant metabolites as candidate diagnostic classifiers. The diagnostic performance of the selected classifiers was then visualized and quantified by the receiver operator characteristic (ROC) curve analysis with a random forest (RF) algorithm. Classifier robustness was estimated by permutation test (permutation times = 1000). Sensitivity, specificity, and accuracy were estimated by 2 × 2 contingency table analysis in Prism 8.0 (GraphPad Software, La Jolla, CA, USA, www.graphpad.com).

## 3. Results

### 3.1. Method Optimization

**Figure 2:**
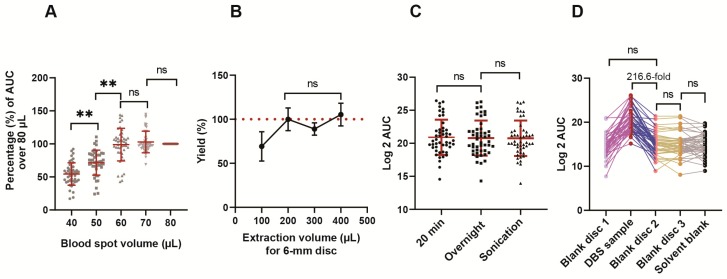
Dried blood spot (DBS) workflow optimization. (**A**) The effect of blood spot volumes. (**B**) The effect of the volumes of the extraction buffer. (**C**) The different extraction methods. (**D**) The effect of cross-contamination between punches. A total of 47 representative metabolites covering the whole gradient were selected for the analysis. Data were mean ± SD. One-way ANOVA followed by Tukey’s test was used. **p < 0.01 and ns indicated non-significant.

We first optimized the blood spot volumes on GE Whatman 903^TM^ protein saver cards. A subset of metabolites (47 compounds), selected to cover the entire LC gradient and all the chemical classes we targeted, was analyzed in more detail. Figure S1 shows the correlation between the spot volumes and the diameters of DBSs. The peak area for 47 representative metabolites increased from 40 to 60 µL of spotted volume. (Figure 2A and Table S2). We found that blood volumes of 60–80 µL spotted on Whatman 903^TM^ cards resulted in an even spreading of the sample and equivalent yields in the punch. For convenience, we used 70 µL for all subsequent experiments. This produced a blood spot diameter of 13.5 ± 0.55 mm. It was calculated that a 6.3 mm diameter hole punch of the dried blood spot contained the dried residue from 13.4 µL of blood (Figure S1B).

We used methanol: acetonitrile: water (MEOH-ACN-H_2_O; 40:40:20) as the buffer for metabolite extraction [21] and optimized the extraction volume for the DBS discs. The yield remained similar with ≥ 200 µL of extraction buffer (Figure 2B). Three different extraction protocols were tested including incubation on ice for 20 min, incubation at 4 °C overnight, and sonication in an ice bath for 5 min. No significant differences between the three extraction methods were identified (Figure 2C).

Cross-contamination during the processing of DBS for metabolomic analysis was minimal. The average peak intensity of the first blank disc following DBS sample was 217-fold lower than DBS sample, which was similar to the second punch of the blank disc and the solvent blank (Figure 2D).

### 3.2. Sample Type Comparisons

**Figure 3:**
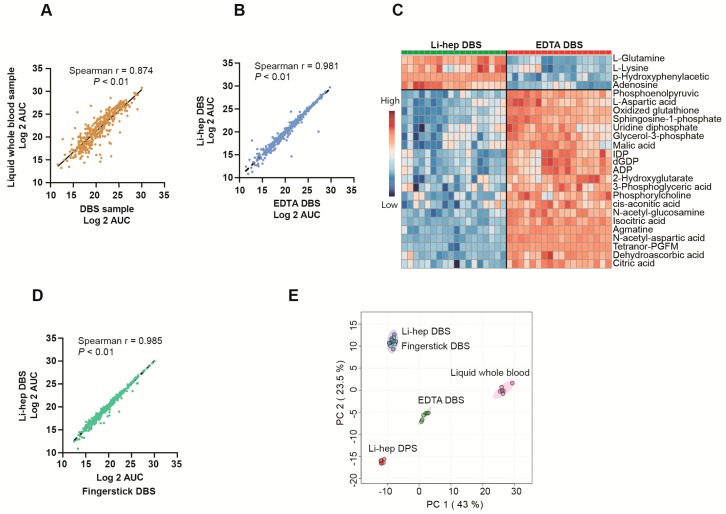
Sample type comparisons. (**A**) The correlations of areas under the curve (AUCs) of metabolites between wet blood and DBS. All 430 detected metabolites were used and the AUCs were log 2 transformed. The Spearman rank correlation was performed. The other correlation analyses in this figure were similarily performed. (**B**) The correlations of metabolite AUCs between Li-hep DBS and EDTA DBS. (**C**) The differentially abundant metabolites between Li-hep DBS and EDTA DBS visualized on the heatmap. The top 25 significant metabolites ranked by the student’s *t*-test were listed. *p* < 0.01. (**D**) The correlations of metabolite AUCs between Li-hep DBS and fingerstick DBS. (**E**) A Principal component analysis (PCA) of the five different sample collection methods showed that samples collected by fingerstick lancet and heparinized venous blood were superimposable.

We next analyzed the similarities and differences between different sample types. A comparison of Li-heparin DBS to Li-heparin liquid whole blood revealed no significant difference in the total number of metabolites detected (S/N > 3) (431 ± 4 vs 433 ± 2, *p* > 0.05). Four hundred and twenty-four (424) detected metabolites overlapped, nine metabolites were only detectable in DBS, and 11 metabolites were unique in the liquid whole blood (Table S3). The AUCs correlated well between DBS and liquid whole blood samples (Spearman r = 0.874, *p* < 0.01, Figure 3A). The similarities and differences between DBS and DPS are described in the supplemental results (Figure S2 and Table S4).

The anticoagulant used in the blood collection tube affected the resulting metabolome of DBS. Overall, the metabolites from Li-heparin DBS were highly correlated with those from EDTA DBS (Spearman r = 0.981, *p* < 0.01, Figure 3B). However, there were also some notable differences: EDTA DBS yielded higher AUCs for nucleotides and organic acids, while, adenosine, glutamine, L-lysine, and p-hydroxyphenylacetic acid were dramatically lower (*p* < 0.05) (Figure 3C and Table S5).

Li-heparin DBS also correlated well with fingerstick DBS (Spearman r = 0.985, *p* < 0.01, Figure 3D). Samples collected as lithium heparin venous blood and fingerstick capillary blood samples collected by lancet and stored as DBS were indistinguishable by principal component analysis (PCA). Volcano plot analysis showed that out of the 424 detected metabolites in our study, only 10 metabolites were significantly different between Li-heparin DBS and fingerstick DBS (Table S6).

### 3.3. One-Year Stability Analysis

**Figure 4:**
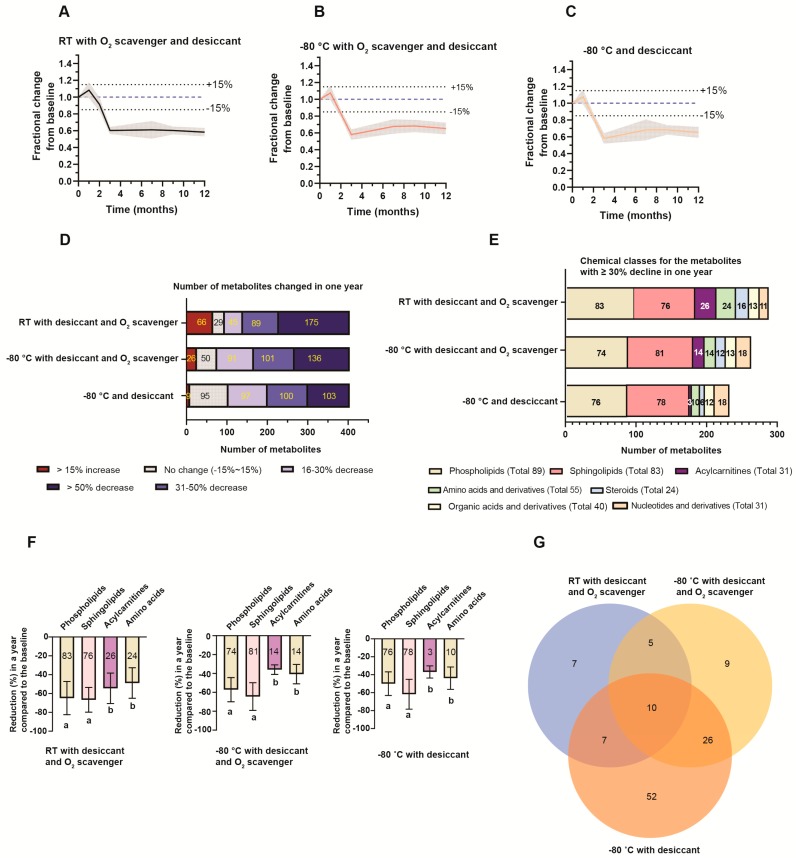
Stability analysis. (**A**–**C**) The fractional change over a year from the baseline for the geomean of all detected metabolites. (**A**) DBS stored at room temperature (RT) with desiccant and an O_2_ scavenger (RT with desiccant and an O_2_ scavenger). (**B**) DBS stored in −80 °C with desiccant and an O_2_ scavenger. (**C**) DBS stored in −80 °C with desiccant. The shaded area represents the standard deviations (SD). (**D**) The number of metabolites altered in a year of storage in different conditions. (**E**) The chemical classes for the metabolites with ≥ 30% decline in one year. The number of metabolites changed was listed in the graph and the total number of targeted metabolites in each chemical class was shown in the legend. (**F**) The percentage of reduction (%) from the baseline for the metabolites in the major chemical classes. The value in the bar graph was the total number of metabolites in each chemical class. A one-way ANOVA followed by Tukey’s test was conducted. Data indexed by different letters (“a” and “b”) were significantly different from each other (*p* < 0.05) in each storage condition. (**G**) The number of stable metabolites over one year of storage in each storage condition. The names of the stable metabolites are listed in Table S7.

We first examined the overall changes in the geometric means of the AUCs for all detectable metabolites over a 1-year period. Based on the analytical variances of our method for DBS (Figure S3), metabolites were considered as stable when the changes were ≤ 15%. Regardless of storage condition, the geometric mean was stable for the first month, rapidly declined to 58%–60% of the baseline by 3 months, and then remained unchanged for the subsequent 9 months (Figure 4A–C). A more detailed analysis, however, revealed differences in individual metabolite stability between the 3 storage conditions (Figure 4D). The best performing condition overall was −80 °C with desiccant and without an O_2_ scavenger (Figure 4D–G).

Surprisingly, the presence of an O_2_ scavenger showed a negative impact on the stability of metabolites stored at −80 °C. The inclusion of an O_2_ scavenger resulted in fewer unchanged metabolites (50 vs 95) (Table S7) and more metabolites with a > 50% decrease (136 vs 103) (Figure 4D and Table S8). The presence of an O_2_ scavenger also caused a subset of metabolites, primarily hydroxyeicosatetraenoic acids (HETEs) and eicosanoids, to have increased AUCs (Figure S4A,B and Table S9). This increase was noted at 1 month and persisted throughout the year of analysis. This increase was most notable at room temperature, but also occurred in the samples stored at −80 °C with an O_2_ scavenger (66 and 26 metabolites, respectively).

Phospholipids, sphingolipids, acylcarnitines, amino acids, and steroids were the main chemical classes with ≥ 30% reduction (Figure 4E). A few examples from each chemical class demonstrating the changes in metabolite abundance within a year at RT with desiccant and an O_2_ scavenger are shown in Figure S4C–L. Interestingly, we noticed that both the number of decreased phospholipids and sphingolipids and the percentage of decline from the baseline were similar in all three storage conditions (Figure 4E,F). In terms of phospholipid subclasses, phosphatidylserine (PS), phosphatidylinositol (PI), phosphatidylethanolamine (PE), Bis(monoacylglycero)phosphate (BMP) and phosphatidylglycerol (PG) had a more significant reduction than cardiolipins (CL), phosphatidic acid (PA), and phosphatidylcholine (PC) (Figure S5A). The saturation of the phospholipid acyl chain had no effect on the stability of phospholipids on DBS cards, and the levels of reduction for sphingolipids were not correlated with acyl chain length (Figure S5B,C).

In contrast, the decline of acylcarnitine and amino acid AUCs from the baseline was decreased at −80 °C compared to room temperature storage (Figure 4E). Out of the 31 acylcarnitines measured, 26 were reduced by 54.5% ± 16.2% at room temperature, but only three acylcarnitines were reduced at −80 °C with desiccant, with an average reduction of 36.9% ± 6.8% (Figure 4F). Similarly, the number of amino acids with ≥ 30% reduction dropped from 24 (out of 55) when the cards were stored at room temperature to 10 for the DBS samples stored at −80 °C with desiccant.

Out of 404 detected metabolites, the number of metabolites that was not significantly altered after one year of storage ranged from 29 metabolites at RT with desiccant and an O_2_ scavenger to 50 at −80 °C with desiccant and an O_2_ scavenger, and 95 at −80 °C with desiccant only (Figure 4D and Table S7). A Venn diagram of the overlapping metabolites between the 3 conditions is shown in Figure 4G. Interestingly, 10 metabolites remained stable at all three conditions. These included creatine, hydroxyproline, leucine, acetoacetic acid, and O-acetylserine (Figure 4G and Table S7).

### 3.4. Clinical Application in MELAS

The average intra-day and inter-day RSDs were 9.7% and 10.9% for controls, and 9.1% and 13.9% for the pooled MELAS samples (Figure S3A–D), well within the limit of 20% for RSDs listed in the Food and Drug Administration (FDA)’s guideline for bio-analytical method validation using LC-MS [23].

**Figure 5:**
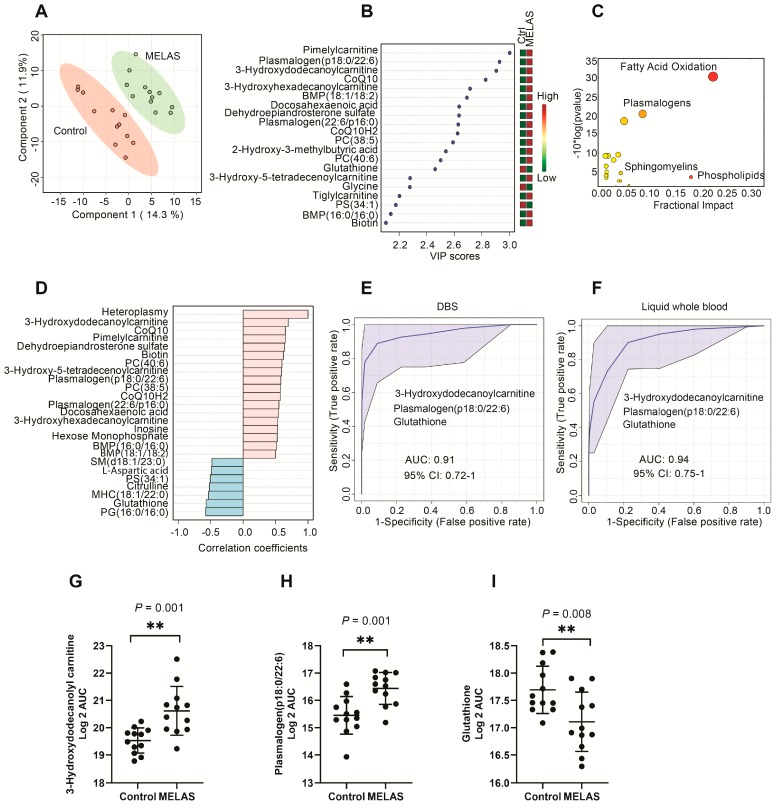
Metabolomic fingerprints and biomarkers in DBS of MELAS patients. (**A**) Partial least squares discriminant analysis (PLS-DA) revealed the complete separation of MELAS patients from the controls (N = 12/group). (**B**) variable importance in projection (VIP) plot of the top 20 metabolites with the highest separation power between the controls and MELAS patients in PLS-DA. A VIP score > 1.5 was considered as statistically significant. (**C**) A pathway analysis of the significantly altered metabolites in the DBS of MELAS patients compared to the controls. The fractional impact of a pathway is defined as the sum of the absolute values of z-scores for the significant metabolites (PLS-DA VIP score > 1.5) divided by the sum of the absolute values of z-scores for all the detected metabolites in that pathway. (**D**) The top 20 metabolites correlated with heteroplasmy. A Spearman correlation analysis was performed. (**E**) A receiver operator characteristic (ROC) analysis to evaluate the diagnostic performance of three selected metabolites in DBS for MELAS. (**F**) The diagnostic performance of the same three metabolites in Li-heparin liquid whole blood for MELAS. Data were log 2 transformed before analysis. (**G**–**I**) Univariate statistical analysis of three selected biomarkers in the DBS of MELAS and controls. A Student’s *t*-test was performed after the log 2 transformation of the data.

The raw DBS metabolomic data for MELAS samples and controls are in Table S10. PLS-DA revealed a clear separation of the blood metabolome of MELAS patients from the controls (Figure 5A). VIP analysis listed the top 20 metabolites with the highest separation power in PLS-DA (Figure 5B). Fatty acid oxidation and synthesis, plasmalogens, phospholipids, and sphingolipids were the major metabolic pathways disturbed in MELAS patients (Figure 5C). Figure 5D shows the metabolites that were significantly correlated with heteroplasmy.

The highest area under the ROC curve was 0.91 (95% CI: 0.72–1) using three metabolites including 3-hydroxydodecanoylcarnitine, plasmalogen (p18:0/22:6), and glutathione (Figure 5E). The sensitivity was 84.6% (95% CI: 54.6–98.1%) and the specificity was 90.9% (95% CI: 58.7–99.8%) (Table S11). Similar diagnostic accuracy, sensitivity, and specificity were obtained in the liquid whole blood from lithium-heparin samples using the same set of metabolites (Figure 5F and Table S11). Univariate statistical analysis showed that 3-hydroxydodecanoyl carnitine and plasmalogen (p18:0/22:6) were significantly higher in MELAS samples than the controls (*p* = 0.001 for both metabolites) (Figure 5G–I). In contrast, reduced glutathione (GSH) was dramatically lower in MELAS compared to the controls (*p* = 0.008).

## 4. Discussion

The objective of this study was the development and optimization of a dried blood spot-based, broad-spectrum targeted metabolomic method. We performed the first systematic optimization of DBS preparation, metabolite extraction and detection, and one-year storage stability in different conditions for over 400 metabolites in DBS. In addition, we examined the similarities and differences of the metabolome from DBS as compared to other types of blood samples.

In DBS preparation, blood volume and hematocrit variations are common challenges. Several sophisticated approaches have been proposed for the estimation and correction of blood volume variations [24,25]. Here, we found that the differences in blood spot volume did not affect our metabolomic analysis if the spot volume was over 60 µL on FDA-approved Whatman 903^TM^ protein saver cards. The mass of metabolites in the 6.3 mm punch remained constant as long as at least 90% of the 12 mm dotted circle on the card was filled. This was true if 60 µL, 70 µL, or 80 µL of blood was applied. The hematocrit effect is another potential obstacle to DBS application [26]. Our results showed that hematocrits between 31% and 50% did not play a significant role as a source of variation in broad-spectrum metabolomics results (Figure S6 and Table S12). Previous studies have similarly shown no apparent impact of hematocrit on the quantification of 25-hydroxyvitamin D3 and cyclosporine A with hematocrits ranging from 20% to 60% [27,28].

Drolet et al. reported the detection of 300 polar metabolites from DBS using the combination of HILIC-MS/MS and GC-MS [13]. Our method routinely detected about 430 metabolites from one DBS sample using a single injection. Moreover, 11 chemical classes were covered in our assay, such as lipids and lipid-like molecules, amino acids and derivatives, organic acids and derivatives, nucleotides and derivatives, and acylcarnitines (Figure S7). To our knowledge, this is the first method that measures such a large number of metabolites with a broad range of chemical classes from DBS using a targeted approach in a single injection.

Our results highlight the importance of using a consistent sample type and collection method for metabolomic studies. DBS samples prepared by Li-heparin or EDTA collection tubes have similar levels of precision and reproducibility compared to liquid whole blood, but show clear differences related to the specific class of metabolites detected. Our lab has previously reported similar metabolomic differences between Li-heparin plasma and EDTA plasma samples [21]. The most striking differences were the significant decrease of phosphorylated nucleotides and an increase in nucleosides such as adenosine in Li-heparin samples compared with EDTA samples.

Regarding differences between capillary and venous blood, little has been described beyond some basic biochemical parameters, such as complete blood counts and hemoglobin [29]. Our analysis showed that samples collected by fingerstick lancet or as Li-heparin venous blood and stored as dried blood spots were superimposable by PCA (Figure 3E). This was true despite the comparison between a precisely measured 70 µL of heparinized whole blood spotted with a pipetman, and fingerstick lancet samples that contained variable volumes of blood. A small number (10 of 424) of the metabolites were found to be different in samples collected by fingerstick and heparinized venous blood (Table S6), but 97.6% (414 of 424) of the measured metabolites were indistinguishable by these two sample collection methods. The choice of any specific sampling method for dried blood spot preparation, e.g., fingerstick lancet, EDTA-whole blood, or Li-heparin whole blood, should be standardized at the beginning and not mixed within any given metabolomic study.

It is critical to evaluate the stability of metabolites in DBS to enable the optimization of processes and the proper interpretation of data. Our results demonstrate that most metabolites were stable for the first month of storage, regardless of condition. Similar results have been reported for the stability of amino acids, acylcarnitines, and vitamin D in DBS stored for one month at room temperature with desiccants [30,31]. Besides these routinely analyzed metabolites, in our study, we expanded the stability analysis to other key metabolites in DBS, and for up to one year of storage. We showed that the decline primarily occurred between 1 and 3 months of storage, then did not decrease further for at least 1 year. Additionally, metabolites in DBS were more stable at −80 °C than those stored at room temperature even though the decline cannot be completely prevented. Ten metabolites were found to be unchanged for the entire year, regardless of the storage condition. Further studies are needed to determine if any of these analytes can be used to normalize for storage time. Our results highlight the need for case and control samples used in comparative metabolomic studies to be matched for sample collection method, storage time, and storage conditions. This study focused on the relative changes in the AUCs of the targeted metabolites compared to the baseline sample and did not attempt to determine the absolute concentration of any single metabolite.

In addition to the decrease in the stability of many metabolites in the presence of an O_2_ scavenger, the finding of an apparent increase in abundance of some metabolites was unexpected. This increase occurred rapidly in the first month, slowly approached a plateau after 3 months of storage, then stabilized (Figure S3A,B). The affected metabolites included arachidonic acid, and several of its metabolites, such as 5-HETE and 5, 6-DHET (Table S9). Since the number of metabolites that increased in the presence of more hypoxic environment was less in the cold (−80 °C) than at room temperature, these results may reflect the residual activity of the enzymes needed to synthesize these molecules. For example, lower oxygen tensions may have resulted in improved preservation of several blood proteins, including enzymes like phospholipase A2 (PLA_2_) needed to release arachidonate from phospholipids, arachidonate 5-lipoxygenase (ALOX), and other lipoxygenases that convert arachidonate to 5-HETE and related oxylipins. After 3 months of storage, no further changes were noted (Figure S3).

In this study, we used our dried blood spot method to examine the metabolome in 12 MELAS patients and 12 matched controls. We identified 3-hydroxydodecanoylcarnitine (C12-OH), plasmalogen (p18:0/22:6), and glutathione (GSH) in DBS metabolome as the potential biomarkers for MELAS. Acylcarnitines are involved in the mitochondrial metabolism of fatty acids. Defects of fatty acid oxidation and the carnitine shuttle system had been observed in inborn errors of metabolism with myopathies including MELAS [32]. Similar to our findings, the circulating hydroxylated long-chain acylcarnitines were shown to be significantly elevated in the plasma of patients with mitochondrial myopathy [33]. Plasmalogens are a subtype of phospholipids characterized by the presence of a vinyl–ether bond at the sn-1 position of the phosphoglycerol moiety. The initial steps of plasmalogen biosynthesis are confined to peroxisomes. Due to their unique structure and composition, plasmalogens are enriched in highly aerobic tissues dependent on mitochondria for energy like brain, heart, muscle, and nerve. Plasmalogens play many crucial roles in cellular functions, including reservoirs for second messengers, working as endogenous antioxidants, as well as the structural components of cellular membranes [34,35]. The aberrant metabolism of plasmalogens had been found in Alzheimer’s disease [36], Parkinson’s disease [37], Niemann-Pick disease [38], and cancers [39]. The role of plasmalogens in mitochondrial disorders remains largely unknown. Mitochondrial disorders are associated with redox imbalance, and GSH is the low molecular weight thiol involved in mitochondrial redox signaling. Decreased levels of peripheral whole blood GSH had been previously reported in mitochondrial disease patients, including Leigh syndrome, electron transport chain abnormalities, MELAS, and mtDNA depletion syndrome [40].

Our study had some limitations. First, we only performed the stability analysis for up to one year under three different storage conditions. The stability beyond one year of storage remains unknown. Second, this study did not include a room temperature condition without desiccant or an oxygen scavenger.

## 5. Conclusions

We describe a reproducible dried blood spot-based, targeted metabolomic workflow that is able to reliably detect about 430 metabolites from a diverse array of chemical classes in a single injection and LC-MS/MS analysis. The DBS metabolome had excellent correlations with other blood sample types. The blood volume and hematocrit variations had no significant impact on the assay for a spot volume > 60 µL and hematocrit > 30%. We found that DBS samples were most stable when stored in −80 °C with desiccants and without an O_2_ scavenger. The median AUC for the extracted metabolites stabilized at about 60% of the baseline value after 3 months and did not decline further for at least 1 year of storage. Additionally, all metabolites measured at the baseline remained quantifiable for at least 1 year. The proof-of-concept study with DBS samples from MELAS patients and contemporaneously collected disease controls demonstrates the practical clinical value of the described methods. The case and control samples used in comparative metabolomic studies need to be matched for the sample collection method, storage time, and storage conditions.

## Supplementary Materials

The following are available online at https://www.mdpi.com/2218-1989/10/3/82/s1, Figure S1: The correlation between the whole blood spot volumes and the diameters of the dried blood spots (DBSs). Figure S2: The similarities and differences between DBS and DPS. Figure S3: Frequency distribution analysis of intra-day and inter-day reproducibility for all detected metabolites in DBS of control and MELAS patients. Figure S4: The changes of the representative metabolites on DBS stored at room temperature with an O_2_ scavenger and desiccant for a year. Figure S5: The effect of the head group, acyl chain length, and saturation on the stability of phospholipids and sphingolipids on DBS. Figure S6: The impact of the varying hematocrit values on the metabolomic profiling of DBS. Figure S7: Chemical classes interrogated by DBS Metabolomics. Table S1: The subjects’ characteristics for MELAS patients and controls. Table S2: The effect of blood spot volumes on the AUCs of 47 representative metabolites. Table S3: Metabolites only detected in either Li-heparin DBS or Li-heparin liquid whole blood. Table S4: The full list of differential metabolites between DBS and DPS identified by volcano plot analysis. Table S5: The full list of differential metabolites between Li-hep DBS and EDTA DBS identified by volcano plot analysis. Table S6: The full list of differential metabolites between Li-heparin DBS and fingerstick DBS identified by volcano plot analysis. Table S7: The list of stable metabolites on DBS after one year of storage at three different conditions. Table S8: The list of metabolites with decreased AUCs after one-year of storage. Table S9: The list of metabolites with increased AUCs after one-year of storage. Table S10: The raw DBS metabolomic data for MELAS study. Table S11: Broad-spectrum targeted metabolomic profiling of DBS and liquid whole blood yields a similar diagnostic accuracy for MELAS. Table S12: The list of metabolites that were not significantly associated with hematocrit changes.
