# Supplementary material for: Improved Dried Blood Spot-Based Metabolomics: A Targeted, Broad-Spectrum, Single-Injection Method
Source: Metabolites. 2020 Feb 27;10(3):82. doi: 10.3390/metabo10030082 (PMC7143494; doi:10.3390/metabo10030082)
Supplement: Supplementary file 1 [file metabolites-10-00082-s001.zip › Supplemental figures.docx]

**Supplemental Figures**

**Improved Dried Blood Spot-Based Metabolomics: A Targeted, Broad-Spectrum, Single-Injection Method**

Kefeng Li^1,2a^, Jane C. Naviaux^1,3^, Jon Monk^2^, Lin Wang^1,2^, Robert K. Naviaux^1,2,4,5,a^

The Mitochondrial and Metabolic Disease Center^1^, Departments of Medicine^2^, Neuroscience^3^, Pediatrics^4^, and Pathology^5^, University of California, San Diego School of Medicine, San Diego, CA

**This file contains**

**Supplemental results**

**Figure S1.** The correlation between the whole blood spot volumes and the diameters of the dried blood spots (DBSs).

**Figure S2.** The similarities and differences between dried blood spots (DBS) and dried plasma spots (DPS).

**Figure S3.** Frequency distribution analysis of intra-day and inter-day reproducibility for all detected metabolites in DBS of control and MELAS patients.

**Figure S4.** The changes of the representative metabolites on DBS stored at room temperature with O_2_ scavenger and desiccant for a year. S1-S3 indicated the samples from 3 subjects.

**Figure S5.** The effect of the head group, acyl chain length, and saturation on the stability of phospholipids and sphingolipids on DBS.

**Figure S6.** The impact of the varying hematocrit values on the metabolomic profiling of DBS.

**Figure S7.** Chemical classes interrogated by DBS metabolomics.

**Supplemental references**

**Supplemental results**

**The comparison between dried blood spots (DBS) and dried plasma spots (DPS)**

We found significant correlations of metabolites between DBS and DPS (r = 0.904 and 0.866, rank based on Spearman method) but correlation coefficients were lower than those of technical replicates for DBS and DPS (DBS_rep1 vs DBS_rep2; DPS_rep1 vs DPS_rep2) (Figure S2A). In regard to the differences between DBS and DPS, volcano plot analysis revealed that 77.4% of metabolites in DBS had significantly higher AUCs than the corresponding metabolites in DPS. A few examples were propionylcarnitine, 12-HETE, NAD, PS (34:1) and PE (32:1) (Figure S2B and Table S4).


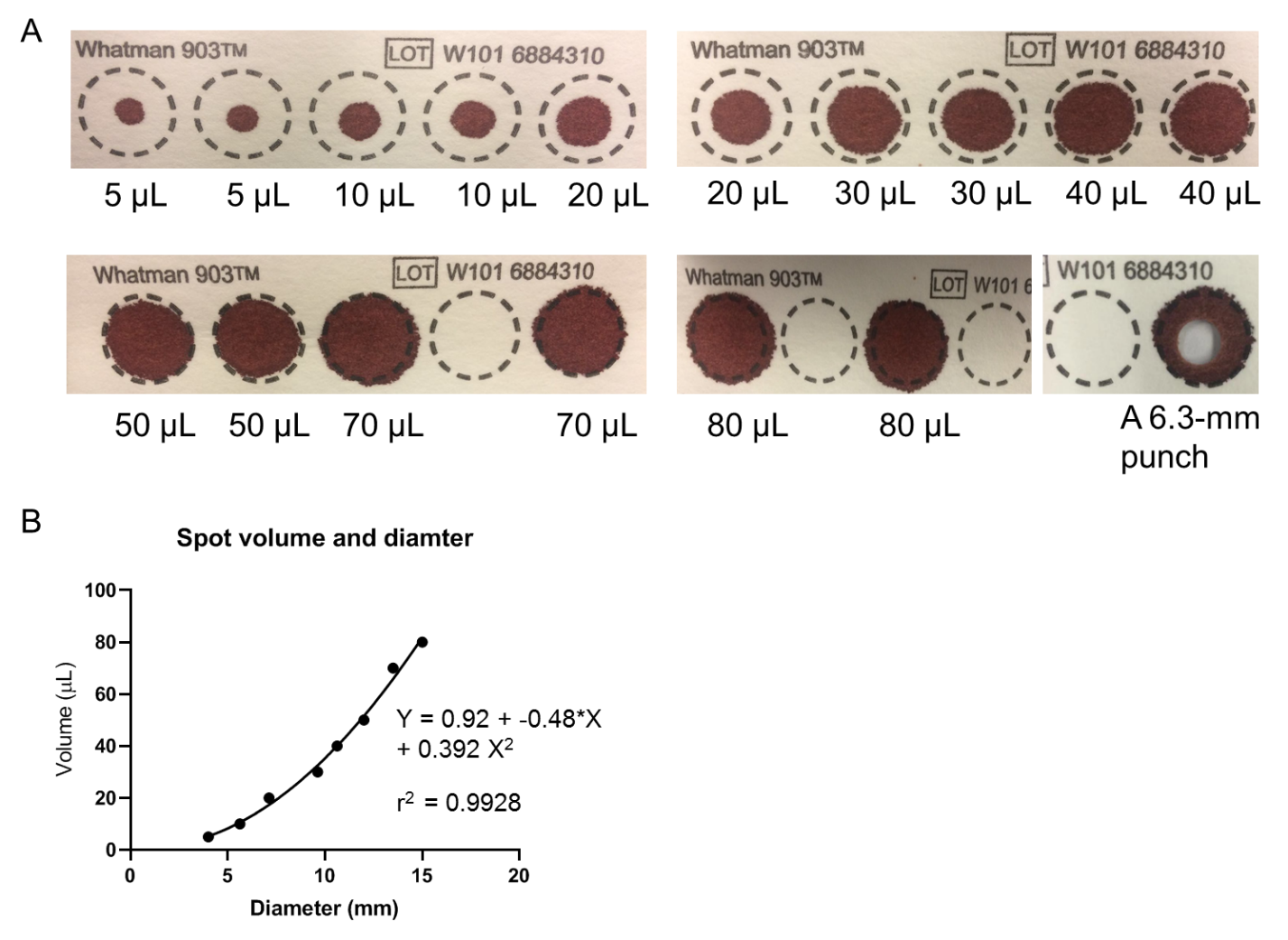


**Figure S1. The correlation between the whole blood spot volumes and the diameters of the dried blood spots (DBSs).** (A), The photos of DBSs with different spot volumes (5 µL – 80 µL). (B), The relationship between whole blood spot volumes and the diameters of DBSs (mm). Second order polynomial regression analysis (quadratic) was performed.

**
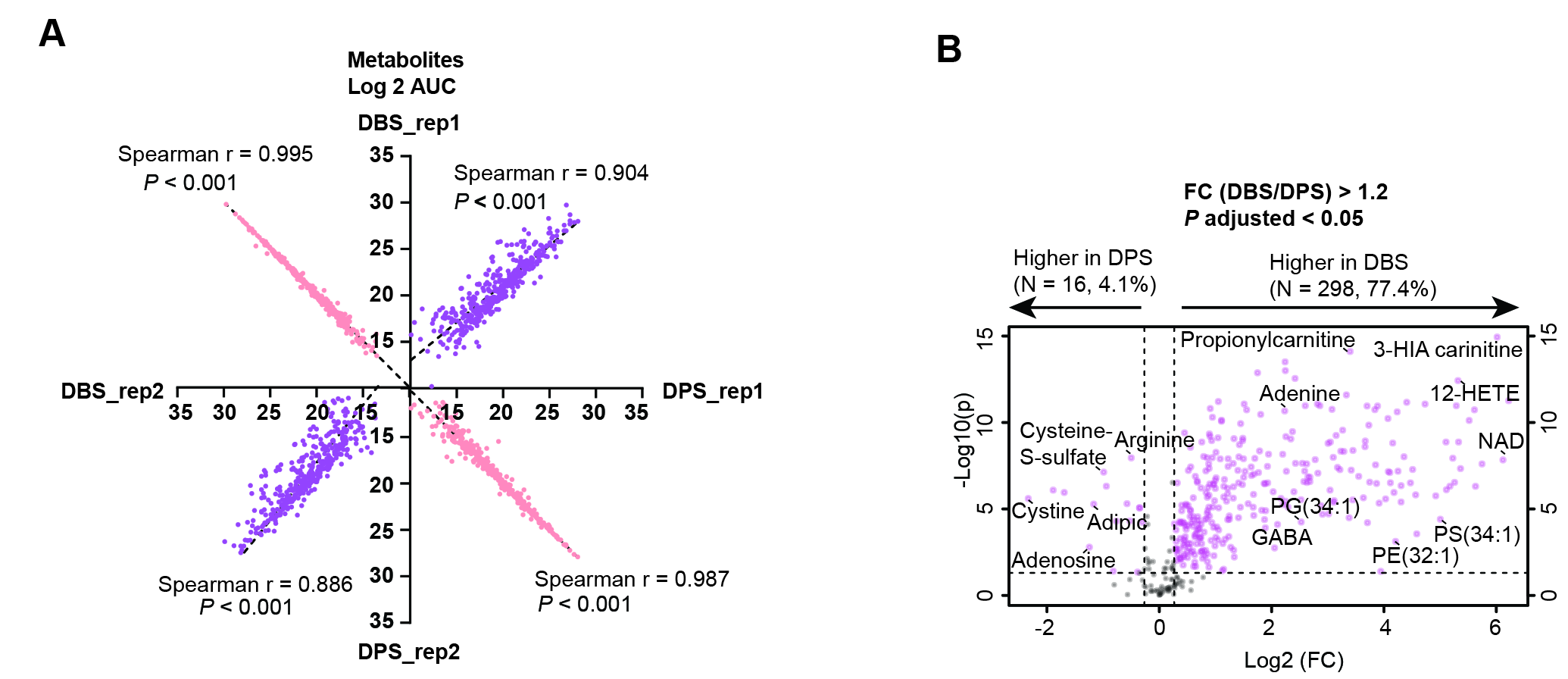
**

**Figure S2. The similarities and differences between dried blood spot (DBS) and dried plasma spot (DPS).** (A), Correlations of metabolites peak area between DBS and DPS. Rep 1 and rep2 were technical replicates. (B), Volcano plot showed the differentially abundant metabolites between DBS and DPS. Fold change (DBS/DPS) > 1.2 and *P* < 0.05.

*
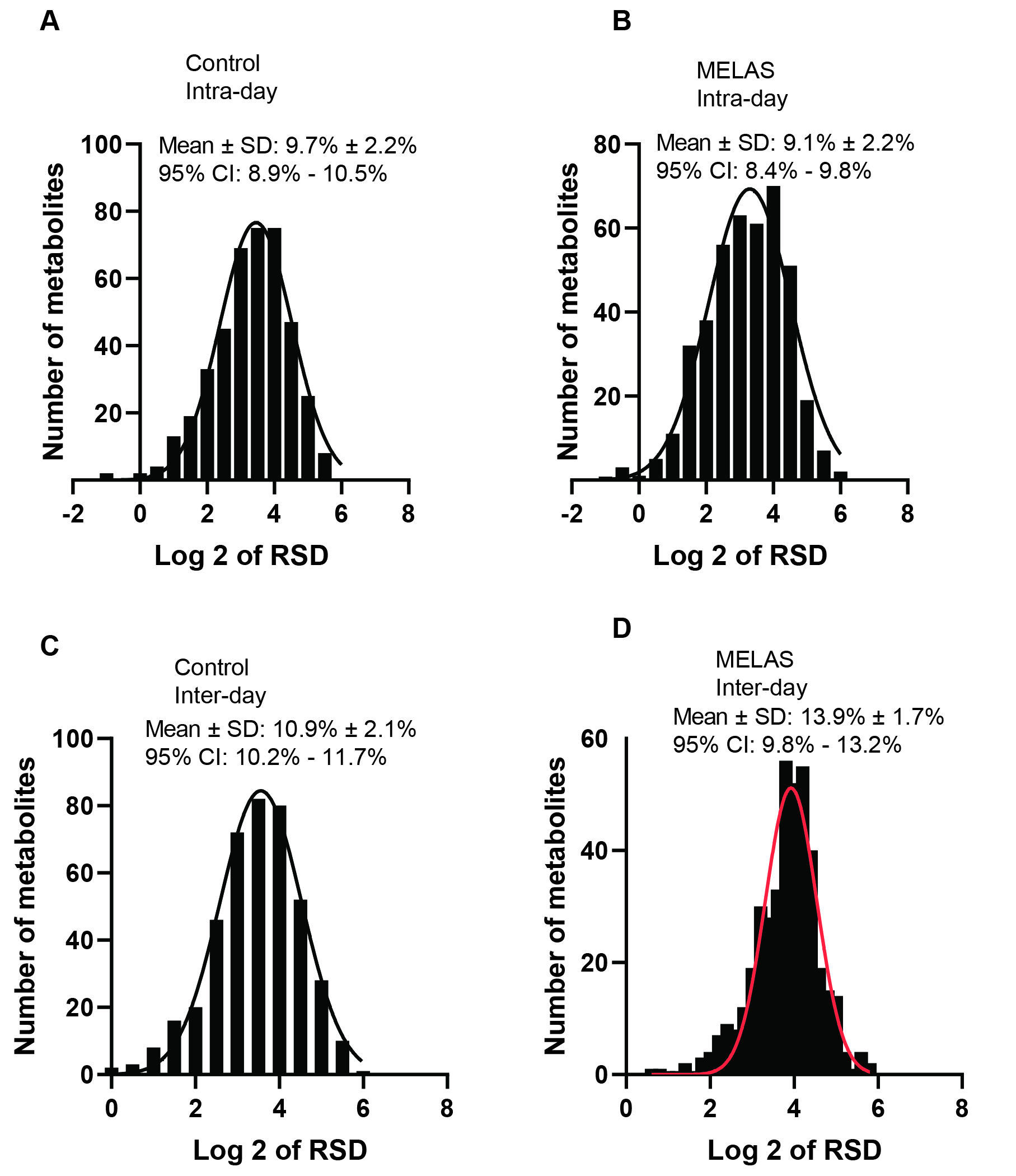
*

**Figure S3. Frequency distribution analysis of intra-day and inter-day reproducibility for all detected metabolites in DBS of control and MELAS patients.** (A), Histogram of intra-day RSDs of metabolites in the pooled control sample. (B), Histogram of intra-day RSDs of metabolites in the pooled MELAS sample. (C), Histogram of inter-day RSDs of metabolites in the pooled control sample. (D), Histogram of inter-day RSDs of metabolites in the pooled MELAS sample. The controls and MELAS samples were pooled. For Intra-day RSDs: the pooled control and MELAS samples were injected 5 times within a day and the RSD for each pool was calculated. For inter-day RSDs: the pooled control and MELAS samples were injected once daily for continuous 5 days. The RSDs of the metabolites across 5 days were calculated. 95% confidence intervals for the log-normally distributed RSDs were calculated as the geometric mean ÷ and x the (antilog)^2^ of the SD calculated in log space [1].

**
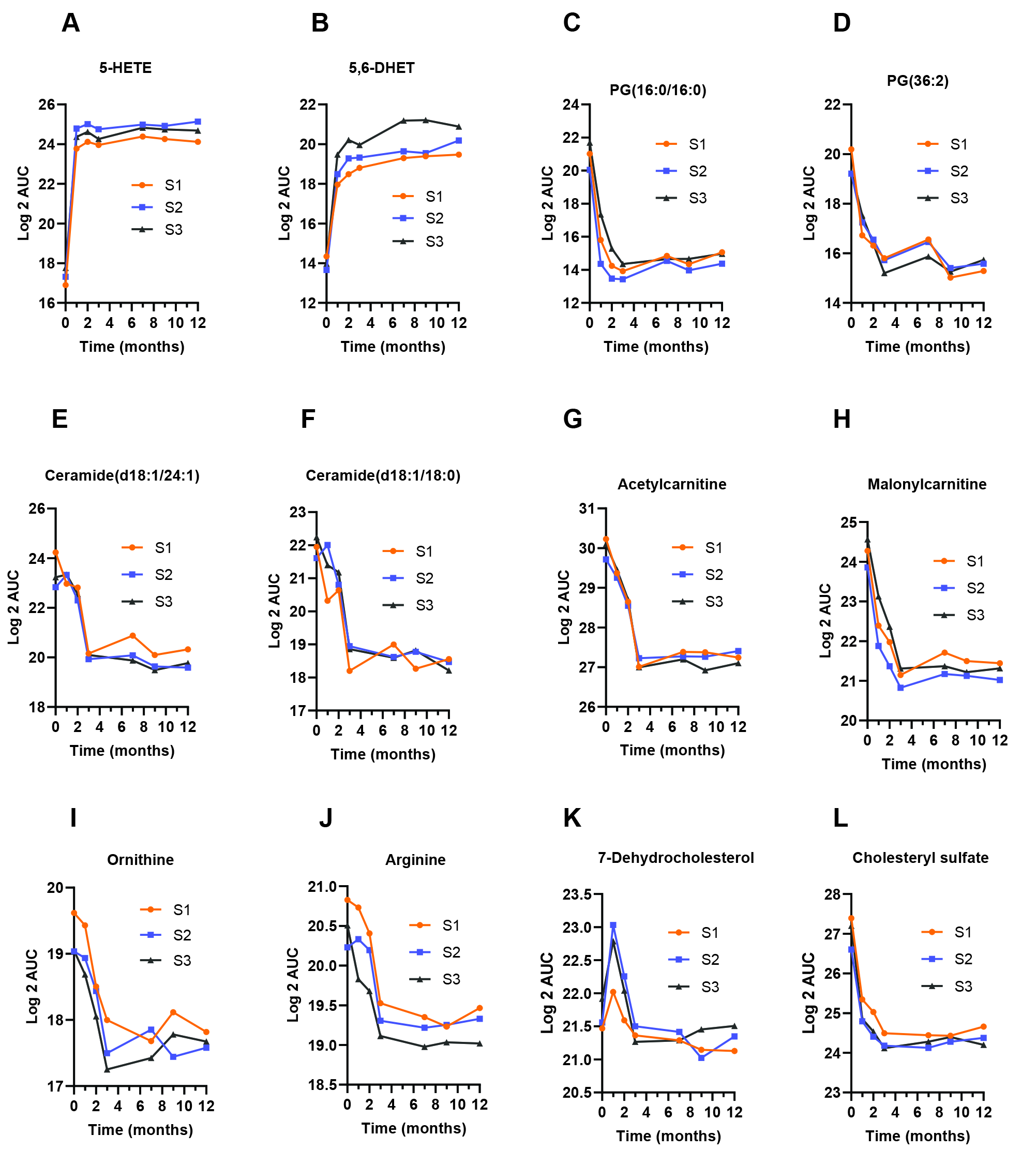
**

**Figure S4. The changes of the representative metabolites on DBS stored at room temperature with O_2_ scavenger and desiccant for a year.** S1-S3 indicated the samples from 3 subjects.


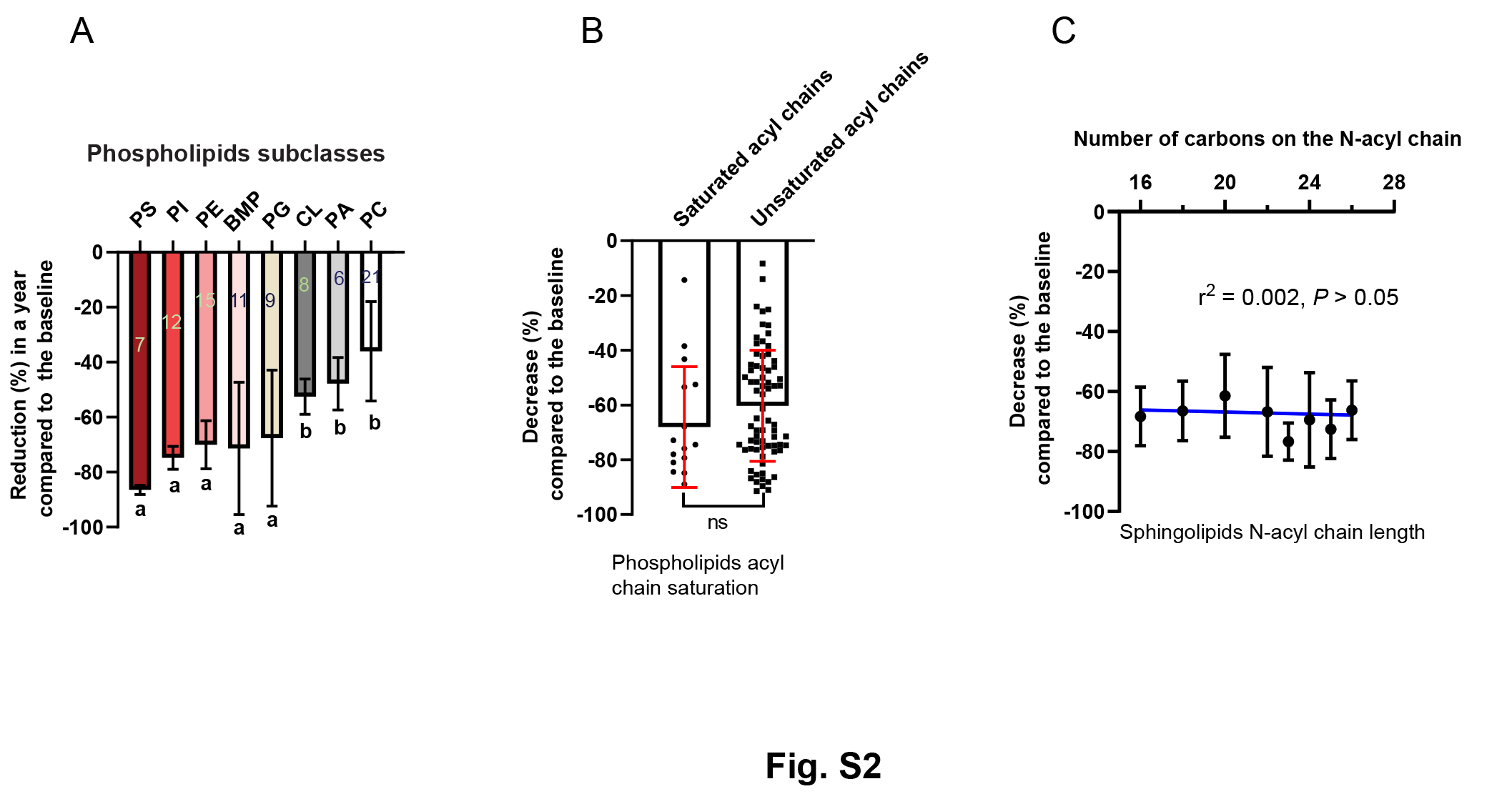


**Figure S5. The effect of the head group, acyl chain length, and saturation on the stability of phospholipids and sphingolipids on DBS**. (A), The percentage of reduction from baseline for the subclasses of phospholipid. The values in the bar graph were the number of metabolites in each subclass of phospholipid we targeted. The columns indexed by different letters are significantly different from each other according to one-way ANOVA followed by Tukey’s test (*P* < 0.05). (B), The comparison of the percentage of reduction for phospholipid between saturated acyl chains (N = 16) and unsaturated acyl chains (N = 72). DBS samples were prepared from 3 subjects and stored at RT with desiccant and O_2_ scavenger for one year. ns: not significantly different (*P* > 0.05). (C), The reduction of sphingolipid is not correlated with the N-acyl chain length. Spearman correlation was conducted (r^2^ = 0.002, *P* > 0.05).

*
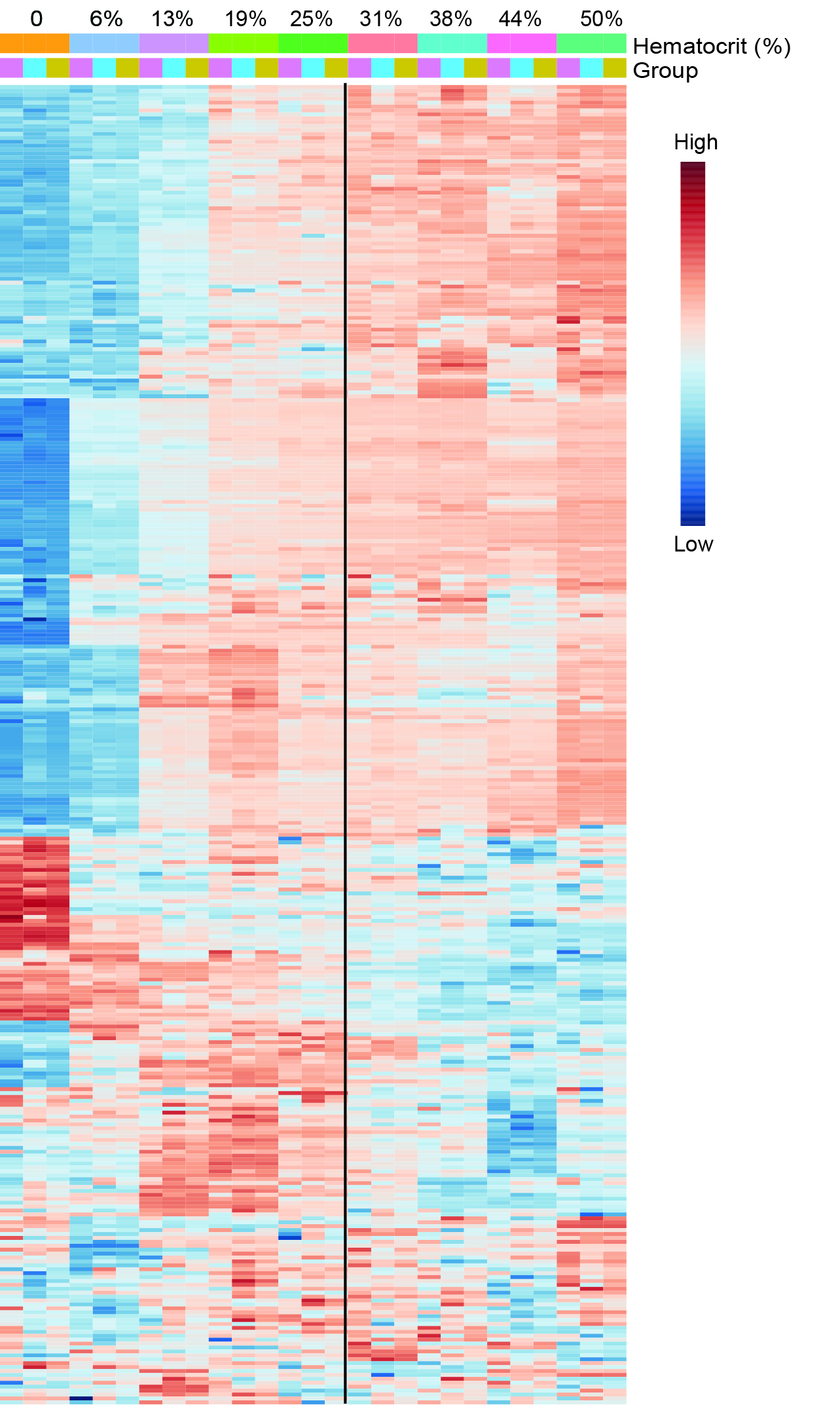
*

**Figure S6. The impact of the varying hematocrit values on the metabolomic profiling of DBS**.

The plasma and whole blood samples from the same individual were mixed to obtain different hematocrit values (0%, 6%, 13%, 19%, 25%, 31%, 38%, 44% and 50%). Seventy µL of the mixture was then spotted on Whatman 903 protein saver cards and dried for 48 h before analysis. Six-mm punches (3 replicates per group) were used for LC-MS/MS analysis as described in the method section.

**Figure S7. Chemical classes interrogated by DBS Metabolomics.** About 430 metabolites in 11 chemical classes were routinely detected from one DBS sample using a single injection.

**Supplemental references**

1. Limpert, E.; Stahel, W.A. Problems with using the normal distribution--and ways to improve quality and efficiency of data analysis. *PloS one* **2011**, *6*, e21403, doi:10.1371/journal.pone.0021403.
